# Predicting Rheological Properties of Asphalt Modified with Mineral Powder: Bagging, Boosting, and Stacking vs. Single Machine Learning Models

**DOI:** 10.3390/ma18122913

**Published:** 2025-06-19

**Authors:** Haibing Huang, Zujie Xu, Xiaoliang Li, Bin Liu, Xiangyang Fan, Haonan Ding, Wen Xu

**Affiliations:** 1 Xinyu Highway Survey and Design Institute, Xinyu 338000, China; 2 Road Material and Structure Engineering Technology Research Center of Jiangxi Provincial, Jiangxi Communications Investment Maintenance Technology Group Co., Ltd., Nanchang 330000, China; 3 School of Transportation and Logistics Engineering, Wuhan University of Technology, Wuhan 430000, China; 340376@whut.edu.cn

**Keywords:** modified asphalt, mineral powder, rheological properties, ensemble machine learning, SHAP

## Abstract

This study systematically compares the predictive performance of single machine learning (ML) models (KNN, Bayesian ridge regression, decision tree) and ensemble learning methods (bagging, boosting, stacking) for quantifying the rheological properties of mineral powder-modified asphalt, specifically the complex shear modulus (G*) and the phase angle (*δ*). We used two emulsifiers and three mineral powders for fabricating modified emulsified asphalt and conducting rheological property tests, respectively. Dynamic shear rheometer (DSR) test data were preprocessed using the local outlier factor (LOF) algorithm, followed by K-fold cross-validation (K = 5) and Bayesian optimization to tune model hyperparameters. This framework uniquely employs cross-validated predictions from base models as input features for the meta-learner, reducing information leakage and enhancing generalization. Traditional single ML models struggle to characterize accurately as a result, and an innovative stacking model was developed, integrating predictions from four heterogeneous base learners—KNN, decision tree (DT), random forest (RF), and XGBoost—with a Bayesian ridge regression meta-learner. Results demonstrate that ensemble models outperform single models significantly, with the stacking model achieving the highest accuracy (*R*^2^ = 0.9727 for G* and *R*^2^ = 0.9990 for *δ*). Shapley additive explanations (SHAP) analysis reveals temperature and mineral powder type as key factors, addressing the “black box” limitation of ML in materials science. This study validates the stacking model as a robust framework for optimizing asphalt mixture design, offering insights into material selection and pavement performance improvement.

## 1. Introduction

With the vigorous development of road construction in China, the frequency of occurrence of asphalt pavement ruts, cracks, and network cracks has increased; thus, higher requirements are placed on asphalt materials [1,2,3,4]. Under the current green environmental protection concept, producing and paving asphalt mixtures at low temperatures is more in line with the needs of the times. As a kind of normal-temperature construction technology, emulsified asphalt mixture has been widely researched and applied due to its energy-saving and environmental protection advantages [5,6]. Nevertheless, emulsified asphalt itself has poor stability and is prone to breakage when mixed with other materials [7]. Thus, many studies focus on the breakage mechanism and performance improvement of emulsified asphalt. Research has found that the physical properties of aggregates are not the main factors affecting the demulsification rate, but the material properties of aggregates, such as acidity, alkalinity, and electronegativity, are the main factors [8].

Mineral powder filler, namely fine aggregate passing through a 200-mesh (75 μm) sieve, is commonly used in asphalt mixtures [9]. A wide variety of mineral powder fillers is used in engineering applications. Traditionally, natural limestone was processed into mineral filler for use in asphalt mixtures. Recently, alternative materials such as waste lime, phosphate waste, dolomite powder, and cement have been investigated as potential fillers in asphalt mixtures.

Mineral powder fillers are also commonly employed as an inert filler in engineering applications; the incorporation of them significantly impacts the mechanical performance of asphalt mortars in pavement systems. Mineral powder in asphalt plays a crucial role in structure formation. In asphalt binders, a certain concentration of mineral powder can significantly reduce the thickness of the asphalt layer on the surface of mineral particles, thereby increasing the structuring degree of asphalt and enhancing the bonding between particles [10]. However, fillers with high specific surface areas may cause sedimentation or flocculation during storage, particularly in cationic emulsified systems, necessitating the use of stabilizers to maintain homogeneity. Additionally, the strict control of filler dosage (typically 2% by weight of water) is required, as overuse may impair ductility and destabilize storage. Emulsified asphalt mixture is a complex system, and individual components or mineral powder properties cannot fully reflect the actual relationship between emulsified asphalt and aggregates [7].

Numerous studies have evaluated the effect of filler type on asphalt mastic’s permanent deformation sensitivity [11,12]. The filler type significantly impacts the complex shear modulus (G*). Dynamic shear rheology (DSR) technology has made significant progress in understanding the interaction between asphalt and filler [13]. In summary, rheological indicators can effectively characterize the interaction between asphalt and filler, but there is less research on emulsified asphalt and mineral powder, especially on emulsified asphalt prepared with different emulsifiers.

Data-driven techniques, like machine learning (ML), deep learning (DL), and big data analytics, have recently gained wide attention in predicting asphalt mixture and pavement performance. Machine learning, a branch of artificial intelligence (AI), is a discipline that enables computer systems to automatically accomplish tasks through experience [14]. Atakan et al. [15] employed random forest (RF) to predict recycled hot-mix asphalt (HMA) performance by integrating aggregate surface area (ASA), aggregate count (NA), and gradation characteristics. The model learned non-linear relationships between aggregate morphology and HMA strength, outperforming traditional empirical models. Sadat et al. [16] developed predictive models for the viscoelastic behavior of modified asphalts using different modifiers based on DSR experimental data. Majidifard et al. [17] proposed innovative ML methods like gene expression programming (GEP) and hybrid artificial neural network/simulated annealing (ANN/SA) to predict the fracture energy of asphalt mixture specimens, highlighting the utility of hybrid models for complex failure mechanisms. AL-Jarazi et al. [18] used SHAP to interpret interlayer shear strength (ISS) predictions from ANN and RF models, identifying aggregate roughness and asphalt viscosity as dominant factors. This addresses the “black box” challenge by linking ML outputs to mechanistic insights. Liu et al. [19] used kernel density estimation to detect outliers in datasets and analyzed the impact of different methods on PG grade encoding. Liu et al. [20] included 7400 samples to train their E* model, avoiding bias toward narrow operational windows. They enhanced the Superpave volumetric mixture design by controlling mixture performance related to rutting. Results demonstrated that XGBoost achieved the highest prediction accuracy among all machine learning models, significantly surpassing Witczak’s equation. Analysis revealed that test conditions and asphalt binder properties were the primary factors influencing E*. ML is excellent at modeling complex non-linear relationships, automating feature importance ranking, and enhancing prediction efficiency through cross-validation [21]. However, ML models rely on high-quality, diverse datasets, and their performance degrades significantly when applied to data outside their training distribution. The black box nature of ML models makes them difficult to interpret mechanistically, so advanced tools such as SHAP are needed to improve explainability.

To reduce errors from bias and variance in different machine learning algorithms, ensemble methods can be used to combine the results of multiple algorithms. Bagging constructs multiple sub-datasets through the bootstrap sampling of the original training data, trains multiple base learners separately on these sub-datasets, and then combines the predictions of these base learners. Random forest is the most typical algorithm based on bagging. Boosting is an iterative algorithm. In each training round, it adjusts sample weights according to the previous base learner’s performance, so that the base learner can focus more on samples mispredicted in the previous round, thereby enhancing model performance step by step. Algorithms like Adaboost and XGBoost are based on boosting [18,20,22]. Stacking uses a meta-learner that blends various basic ML models to enhance accuracy [23,24,25]. The stacking process has two stages: first, train multiple models and combine each model’s output or prediction into a new dataset; second, apply a “meta-learning algorithm” to generate the final result [26].

This study aims to address the complexity of predicting the rheological properties of mineral-powder-modified asphalt. Traditional single models are insufficient in characterizing the interfacial effects between emulsified asphalt and mineral powder, especially in predicting responses under coupled temperature and frequency effects. We compared the predictive performance of single models with three ensemble learning models: bagging, boosting, and stacking. This study involves conducting a series of laboratory experiments. These include preparing emulsified asphalt with cationic emulsifiers (MQ65 and EM520), modifying it with three mineral powders (limestone, diabase, and granite), and using DSR to measure its rheological properties. We systematically gathered key parameters like emulsifier type, mineral powder type, temperature, and loading frequency to build a comprehensive feature matrix. Using rheological response data such as complex modulus (G*) and phase angle (δ) measured by DSR, we established multiple machine learning predictive models. We optimized meta-learner parameters through K-fold cross-validation to enhance model generalization and prediction accuracy. This approach may solve traditional single-model problems in characterizing emulsified asphalt–mineral powder interfacial effects and responding to temperature–frequency coupling.

## 2. Materials and Methods

### 2.1. Raw Materials and Sample-Making

#### 2.1.1. Preparation of Emulsified Asphalt

To emulsify the asphalt, first start and calibrate the PHS-25 meter, which is produced by INESA Analytical Instruments Co., Ltd. in Shanghai, China. Then, prepare a soap solution consisting of 2000 g of water, an emulsifier, hydrochloric acid, and a stabilizer. In this study, MQ65 and EM520 emulsifiers were used at a typical dosage of 2% relative to the water content. Their physical properties are detailed in Table 1. The water temperature (40 °C) and pH (2) must be strictly controlled. Using warm water enhances emulsifier dispersion and reduces pH variation. Mix SBR latex with the soap solution at a dosage of 4% and inject it into the colloid mill’s soap tank. Heat the base asphalt and inject it into the asphalt tank. Control the flow rates of the soap and asphalt to achieve a solid content of 60% in the emulsified asphalt.

#### 2.1.2. Preparation of Emulsified Asphalt Residue

Although numerous standard methods exist for obtaining emulsified asphalt residue, there is no consensus on procedures for removing water from asphalt emulsions, particularly for recovering residue from polymer-modified emulsions. Excessive evaporation or distillation temperatures, along with prolonged heating times, may cause significant polymer degradation, compromising the accuracy of residue property measurements. Consequently, this study adopts a low-temperature evaporation method to prepare the residue of modified emulsified asphalt. The preparation method and instruments are detailed in the referenced literature [27].

#### 2.1.3. Mineral Powders

The LS-POP laser particle size analyzer from Zhuhai OMEC Instrument Co., Ltd. in Zhuhai, China, was used to analyze the particle size of various mineral powders. Before particle size analysis with the laser particle size analyzer, the powders were ultrasonically dispersed in a water solution. Table 2 shows the particle size test results for limestone (LS), diabase (DB), and granite (GN) powders.

### 2.2. Test Methods

The DSR tests were conducted using the KINEXOS DSR instrument from Malvern Instruments Co., Ltd. in Malvern, UK, with a 25 mm diameter parallel plate and 1 mm gap. The testing conditions included a temperature range of 40 °C to 72 °C (at 10 rad/s loading frequency) and a loading frequency range of 0 to 100 rad/s (at 64 °C). Rheological parameters such as complex modulus (G*) and phase angle (δ) were measured to analyze the material’s rheological properties. By conducting strain scanning tests through oscillation, observe the yield point of asphalt (the point where the modulus begins to decrease) and determine the linear viscoelastic region. The control strain level of 0.5% was determined through experiments. The experimental materials include asphalt and asphalt with added mineral powder.

## 3. Methods

### 3.1. Data Preprocessing

Data preprocessing is a set of techniques that convert raw data into high-quality data suitable for machine learning models. Popular data preprocessing steps include cleaning, integration, reduction, and transformation. In this study, the local outlier factor (LOF) algorithm was first used to calculate the local density deviation of data points relative to their neighbors, and samples with much lower density than their neighbors were identified as outliers [26]. After cleaning, the data were transformed. Data transformation is the process of changing data from one format, structure, or value to another. Categorical variables are hard for machine learning models to recognize directly and should be converted to continuous variables. Dummy coding was used in this study, which converts a categorical variable with k categories into k-1 binary variables (dummies).

### 3.2. ML Models

Machine learning is categorized into classification and regression problems and falls into four types: supervised, semi-supervised, unsupervised learning, and reinforcement learning. In supervised learning, estimators are trained to uncover the predictive relationship between independent variables to the target variable. This study aims to predict asphalt content by analyzing temperature, frequency, mineral powder properties, and emulsifier characteristics, making it a regression problem addressed by supervised learning. The machine learning models employed for dataset processing in this study include KNN, BR, DT, the bagging-based RF model, the boosting-based XGBoost model, and the stacking model.

#### 3.2.1. K-Nearest Neighbors Regression (KNN)

KNN is a non-parametric regression approach. For a sample point to be predicted, KNN regression identifies the K most similar (nearest) neighbors in the training dataset. Various distance metrics, such as Euclidean, Manhattan, and Minkowski, can be used, with the Euclidean distance function shown in Equation (1) being the most prevalent.

(1) $$d=\sqrt{\sum_{i}^{n}{\left(x_{i}-y_{i}\right)}^{2}}$$

In the equation, *d* represents the distance between two points *x* and *y*, n represents the spatial dimension, *x_i_* represents the *i*-th coordinate of point *x*, and *y_i_* represents the *i*-th coordinate of point *y*.

Then, the target value of the new sample point is predicted based on the target values of these K neighbors, typically by calculating the average (or a weighted average based on distance) of the K neighbors’ target values to obtain the prediction result.

#### 3.2.2. Bayesian Ridge (BR)

BR is a Bayesian-based linear regression model for regression tasks. It estimates model parameters by introducing prior distributions, automatically addressing overfitting, and providing parameter uncertainty estimates.

#### 3.2.3. Decision Tree (DT)

DT performs classification or regression tasks by recursively partitioning datasets and building tree structures. The DT algorithm follows these steps:

1. Select an initial dataset as the root node.
2. Evaluate each feature’s splitting effect and choose the optimal one.
3. Split the dataset into subsets based on the splitting feature’s values, creating child nodes.
4. Repeat steps (ii) and (iii) for each child node until the stopping criteria are met.
5. Prune the DT by removing unnecessary nodes or branches to enhance generalization.

#### 3.2.4. Random Forest (RF)

RF is a machine learning algorithm based on ensemble learning, constructing a strong model by combining multiple weak models. Its predictions are aggregated via “majority vote” (for classification) or “averaging” (for regression). Each decision tree is trained on randomly selected samples (via Bootstrap sampling) and random feature subsets. This randomness boosts model diversity and effectively reduces overfitting risk.

#### 3.2.5. Extreme Gradient Boosting (XGB)

XGB is an efficient and flexible gradient-boosting framework. Based on the gradient boosting framework, it optimizes the objective function by building multiple weak models (typically decision trees) sequentially, with each tree aiming to correct the prediction errors of the previous one. XGBoost introduces regularization terms into the objective function to control model complexity and prevent overfitting. 

### 3.3. Stacking

**Figure 1:**
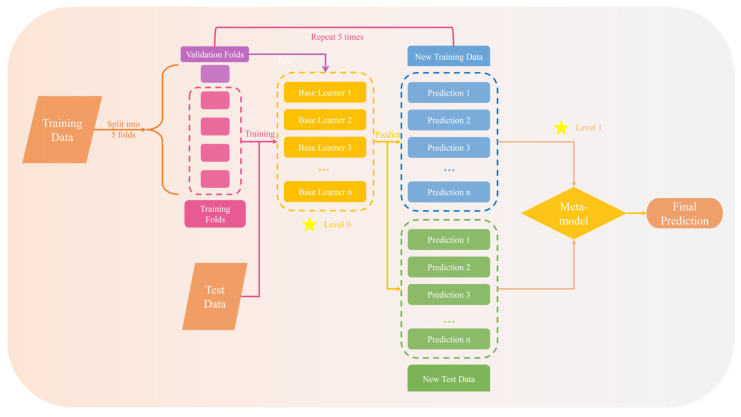
Flowchart of the stacking algorithm.

Stacking is a versatile approach in which a learner is trained to combine the predictions of multiple learners. The individual learners serve as first-level learners, while the combiner is a second-level or meta-learner [26,28]. The conceptual model of stacking is depicted in Figure 1.

Level 0—Using the same dataset to train several models and then generating predictions.

Level 1—Aggregate the predictions generated by many models to obtain the final output.

Stacking is an efficient ensemble algorithm that uses predictions generated by multiple first-level ML algorithms as inputs for second-level algorithms. Second-level ML algorithms are trained to produce more accurate predictions. In stacking algorithms, the training set, fitted by base learners as input for the meta-learner, can cause information leakage and overfitting. Thus, this study improves traditional ensemble algorithms by using cross-validation (CV) in the first-level base learner construction.

Given the original dataset *S* = (*y*, *x_i_*) (*i* = 1, 2, 3, ...), where *y* is the target value and *x_i_* is the *i*-th input feature, *x_i_* is randomly divided into *k* + 1 folds for cross-validation. *S_1_*, *S_2_*, ..., *S_k_* are defined as the training datasets, and *S*_*k*+1_ is the test dataset for k-fold cross-validation. This study uses *K* = 5. The first-level ML algorithms are *B_1_*, *B_2_*, ..., *B_n_*, and the second-level ML algorithm is *M*. *B_n_* is trained on *S_1_* to *S_k_* and predicts *S_k+1_* for each input feature *x_i_*. *B_n_*(*x_i_*) denotes the output of the *n*-th base learner, and *Y* represents the meta-learner’s prediction.

(2) $$Y=M\left(B_{1}\left(x_{i}\right),\ldots ,B_{k}\left(x_{i}\right),\ldots ,B_{n}\left(x_{i}\right)\right)$$

Each base learner predicts the label column of the training and test datasets in K-fold cross-validation. The predicted data for the training and test sets are *Tr* and *Te*.

(3) $$\left.B_{i}\to \left(\begin{array}{c} \vdots \\ Tr_{i}\\ \vdots \end{array}\right.\right)\left(\begin{array}{c} \vdots \\ Te_{i}\\ \vdots \end{array}\right)$$

For base learners 1 to n, n sets of training (*Tr*) and test (*Te*) data were obtained. *Tr_1_* to *Tr_n_* were combined into a new *Tr*, and the same was done for *Te*. The meta-learner of the stacking algorithm was trained on *Tr* and predicted *Te* to obtain the final output *Y*.

(4) $$\left.\left(\begin{array}{cccc} \vdots & \vdots & & \vdots \\ Tr_{1} & Tr_{2} & \ldots & Tr_{n}\\ \vdots & \vdots & & \vdots \end{array}\right.\right)\overset{\mathrm{train}}{\Rightarrow }M\overset{\mathrm{predict}}{\Rightarrow }\left(\begin{array}{cccc} \vdots & \vdots & & \vdots \\ Te_{1} & Te_{2} & \ldots & Te_{n}\\ \vdots & \vdots & & \vdots \end{array}\right)\overset{\mathrm{output}}{\Rightarrow }\left(\begin{array}{c} \vdots \\ Y\\ \vdots \end{array}\right).$$

### 3.4. Evaluation Indicators

Evaluation metrics that are widely used in the machine learning (ML) field were selected to adequately describe model behavior and achieve more intuitive comparison effects [29]. Each evaluation metric has its strengths and limitations. In this paper, four commonly used statistical metrics, namely mean absolute error (*MAE*), mean absolute percentage error (*MAPE*), root mean square error (*RMSE*), and the coefficient of determination (*R*^2^) were used to experimentally evaluate the proposed stacking model. This assesses the prediction behavior and calculates the fit between predicted and actual values.

(5) $$R^{2}=1-\frac{\sum_{i}^{n}{\left(y_{i}-\hat{y_{i}}\right)}^{2}}{\sum_{i}^{n}{\left(y_{i}-\bar{y}\right)}^{2}}$$

*R*^2^ is the coefficient of determination, ranging from 0 to 1, which reflects the goodness of model fit [30].

(6) $$RMSE=\sqrt{\frac{\sum_{i=1}^{n}{\left(\hat{y_{i}}-y_{i}\right)}^{2}}{n}}$$

*RMSE* is the standard deviation of prediction errors, facilitating gradient calculations. It uses mean error and is more sensitive to outliers. When a data point in the learning process is extremely unreliable, the *RMSE* score will be affected [31].

(7) $$MAE=\frac{1}{n}\sum_{i=1}^{n}|y_{i}-\hat{y_{i}}|$$

*MAE* calculates the average magnitude of errors, giving equal weight to each error. It is 0 only when the predicted value exactly equals the true value [32].

(8) $$MAPE=\frac{100\%}{n}\sum_{i=1}^{n}\frac{\left|y_{i}-\hat{y_{i}}\right|}{y_{i}}$$

*MAPE* considers not only the difference between actual and predicted values but also the percentage of error relative to the actual value. This effectively normalizes the error at each data point, reducing the impact of individual outliers on the absolute error [33,34,35].

Low values of *RMSE*, *MAPE*, and *MAE*, and a high value of *R*^2^ indicate the good prediction accuracy of the model. 

In the above equations, $y_{i}$ represents the actual value, $\hat{y_{i}}$ denotes the predicted value, $\bar{y_{i}}$ is the mean value, and n is the number of samples in the dataset.

### 3.5. K-Fold Cross-Validation and Bayesian Optimization

This study systematically optimized machine learning model hyperparameters using a combination of K-fold cross-validation and Bayesian optimization. K-fold cross-validation evaluates generalization by iterative training on K-1 folds and validating on the remaining fold. To balance bias and variance in supervised learning, precise hyperparameter tuning is critical, as these parameters control model complexity. Traditional grid/random search methods are inefficient for high-dimensional datasets, so Bayesian optimization was employed to iteratively update the posterior distribution of the objective function and identify optimal hyperparameters. Hyperparameter combinations were evaluated using the average *R*^2^ from K-fold validation, and final accuracy was tested on a held-out dataset using metrics in Equations (5)–(8). This approach ensures both optimal hyperparameters and practical generalization. Notably, hyperparameter optimization was restricted to the meta-learner to mitigate overfitting.

### 3.6. Shapley Additive Explanations (SHAP) Analysis

SHAP is a powerful tool for interpreting the predictions of machine learning models by calculating the marginal contribution of each feature to the model output. Therefore, in this study, SHAP is used to identify the key feature variables that affect G* and δ. This analysis helps in understanding the importance and impact of different base learners in the stacking model. SHAP values provide insights into how each base learner contributes to the final prediction, which is crucial for model optimization and validation. The definition of SHAP values is shown in Equation (9).

(9) $$\emptyset_{p}=\underset{S\subseteq \left\{x_{1},\ldots ,x_{p}\right\}\left\{x_{p}\right\}}{\sum }\frac{|S|!(P-|S|-1)!}{P!}\left(f\left(S\cup \left\{x_{p}\right\}\right)-f(S)\right)$$

In the formula, ∅*ₚ* represents the SHAP value of feature *p*, *S* is a subset of the model’s features, *xₚ* is the value vector of feature *p*, *p* denotes the number of features, and *f(S)* is the prediction based on the feature values in subset *S*.

## 4. Results and Analysis

### 4.1. Comparison of Model Prediction Performance

In this study, we developed three base learner models and three ensemble learning models to predict the rheological properties of mineral powder-modified asphalt. The three base learner models were BR, KNN, and DT. The three ensemble learning models included the bagging model represented by RF, the boosting model represented by XGB, and the stacking model. For the stacking model, the base learners were KNN, DT, RF, and XGB, while the meta-learner was BR.

Based on the results presented in Table 3, it is evident that the stacking model offers significant advantages for predicting the rheological properties of mineral powder-modified asphalt. For G*, while the *R*^2^ values of the RF and XGB models are higher than those of the DT model, and their *MAE*, *MAPE*, and *RMSE* values are lower than those of the DT model, indicating the superiority of ensemble learning models, the stacking model not only maintains relatively high fit goodness but also exhibits excellent generalization ability. This is crucial for ensuring that the model performs well on unseen data, which is a key requirement in practical applications.

For δ, the RF and XGB models also outperform the DT model in terms of *R*^2^, *MAE*, *MAPE*, and *RMSE*, demonstrating the effectiveness of ensemble learning approaches. Moreover, the stacking model comprehensively outperforms both RF and XGB models while maintaining good generalization ability. This indicates that the stacking model is not only accurate in fitting the training data but also robust in predicting new, unseen data.

### 4.2. Prediction

**Figure 2:**
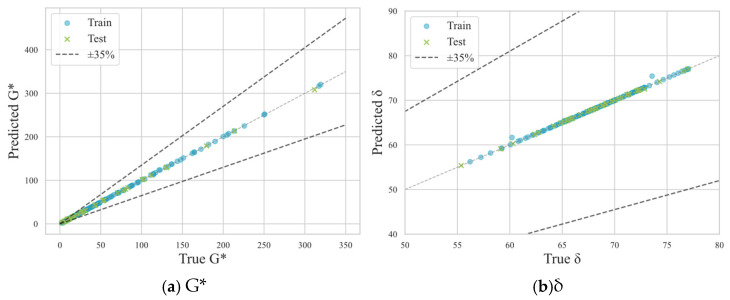
Comparison of measured and predicted values by the stacking model for (**a**) complex shear modulus and (**b**) phase angle.

Figure 2 illustrates the high prediction accuracy of the stacking model when dealing with unseen data, revealing a significant correlation between the predicted and true values of G* and δ for both the training and test sets. The data points are closely aligned with the 45-degree line, indicating that the model’s predictions are highly accurate. Additionally, the dashed lines representing the ±35% error range show that the whole predictions fall within this acceptable error margin, further highlighting the model’s precision.

**Figure 3:**
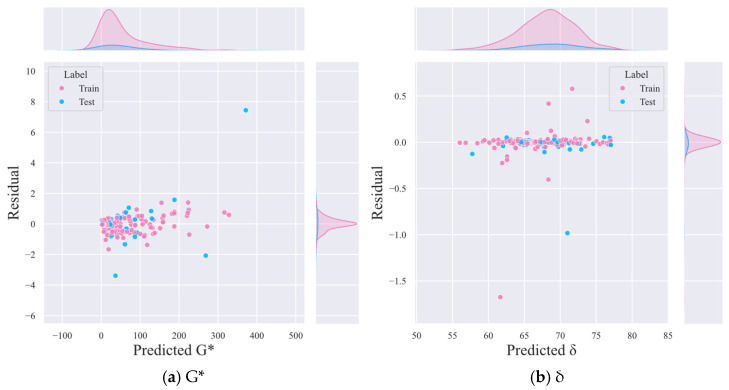
Distribution of prediction errors by the stacking model for prediction of (**a**) complex shear modulus and (**b**) phase angle.

Figure 3 provides a residual analysis for both G* and δ, showing that the residuals are evenly distributed and centered around zero. The density plots on the sides of the residual plots indicate a high density of zero error, suggesting that the model’s prediction errors are minimal. This uniform distribution of residuals and the high density of zero errors demonstrate the reliability of the model’s predictions, confirming that the stacking model is capable of producing consistent and accurate results across different datasets.

### 4.3. SHAP Analysis

#### 4.3.1. Base Learners

**Figure 4:**
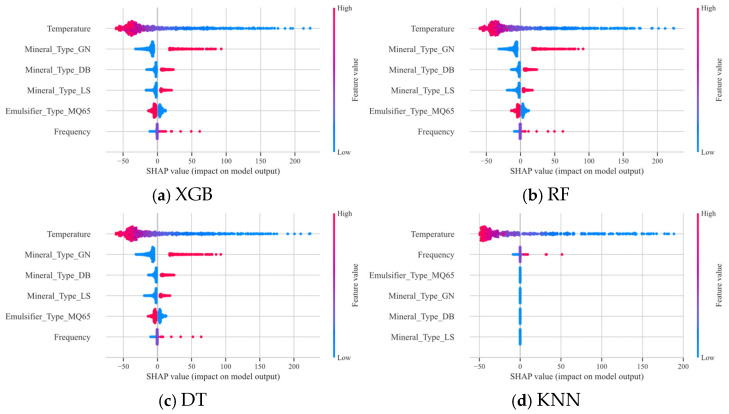
SHAP contribution analysis for the base-learners in the first-level G* model.

As shown in Figure 4, the four base learners XGB, RF, DT, and KNN all indicate that temperature has the greatest impact on G*, with an increase in temperature leading to a decrease in G* and a weakening of the resistance to deformation. Mineral powder, an important component of asphalt mortar, is typically used to improve the high-temperature stability of asphalt mixtures. It enhances the stiffness of the entire system by filling and strengthening the asphalt matrix. However, an increase in temperature intensifies the mobility of the molecular chains in the asphalt, weakening the stability of the network structure formed between the mineral powder and the asphalt. In addition, the decrease in the thickness of the asphalt film around the ore powder particles may lead to an increase in the direct contact between the ore powder particles, reducing the contribution of the ore powder to the overall stiffness, further resulting in a decrease in G*.

The addition of various mineral powders significantly enhances the G* of emulsified asphalt mastics compared to emulsified asphalt residues, indicating that mineral powders can considerably increase the G* of emulsified asphalt. The consistency in the complex shear modulus among the mastics of the three types of mineral powders and emulsified asphalt is evident. The ranking of G* enhancement is as follows: granite (GN) > diabase (DB) > limestone (LS). This demonstrates that granite exhibits the most significant effect on improving the G* of emulsified asphalt.

The type of modifier and frequency have negligible effects on G*. In contrast to the results demonstrated by the other three models, the KNN model exhibits discrepancies, which suggests that the issue may lie within the KNN model itself.

**Figure 5:**
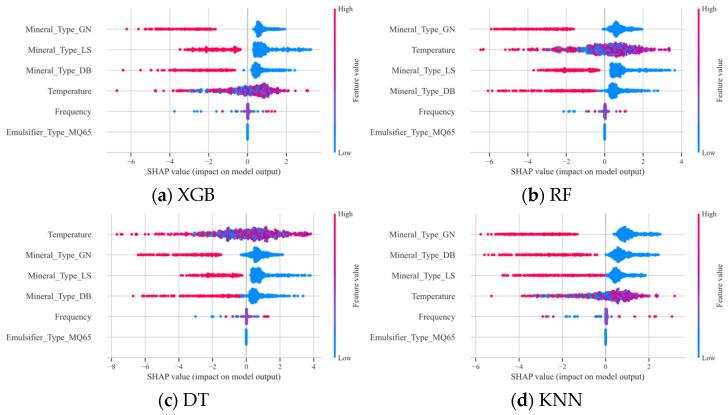
SHAP contribution analysis for the base-learners in the first-level δ.

As shown in Figure 5, adding mineral powder significantly affects the phase angle. The ranking of G* enhancement is as follows: granite (GN) > diabase (DB) > limestone (LS). The addition of mineral powder reduces the phase angle. Mineral powder particles fill the voids in the asphalt binder, reducing the amount of free space within the material and creating a more compact structure. This results in a stiffer and less viscous asphalt binder, reflected in a decreased phase angle. The addition of mineral powder also increases the number of particle contacts within the asphalt binder, creating friction that restricts the movement and flow of the material. Mineral powder particles act as reinforcing agents, effectively transferring stress from the asphalt matrix to the particles. Since mineral powder particles have a higher modulus than the asphalt binder, they can bear part of the load and prevent excessive deformation of the asphalt binder. The impact of temperature should not be overlooked, as its effect on the phase angle shows no clear trend. This may be due to the complex interactions of various components in the modified asphalt. An increase in frequency leads to an increase in the phase angle, as the material’s response becomes more viscous at higher frequencies, reflected by the increased phase angle. Similarly to G*, the type of modifier has almost no effect on the phase angle.

#### 4.3.2. Meta-Learner

The meta-learner (Bayesian ridge) generates the final prediction of the stacking model based on the outputs of the base learners. The importance of the input features to the meta-learner, which are the prediction results of the base learners, can be analyzed to interpret the stacking model indirectly.

**Figure 6:**
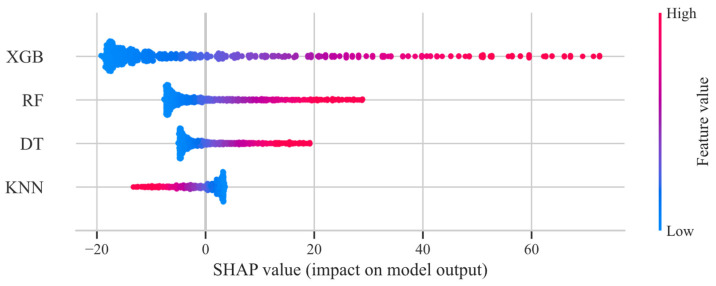
SHAP contribution analysis for the meta-learner in the second-level G*.

For G*, as shown in Figure 6, the SHAP values of XGB exhibit the widest distribution range, from −20 to 70. This indicates that XGB has the most significant impact on the model output, with notable positive and negative contributions across different samples. 

The SHAP values of RF are concentrated around 0, suggesting that its contribution to the final prediction is relatively stable, but its overall influence may be weaker compared to XGB. The complementarity of XGB and RF is also noteworthy. The SHAP value distribution of XGB covers both positive and negative ranges, while RF’s SHAP values are concentrated in the middle range. This suggests that the two models may complement each other in complex samples, with XGB handling extreme values and RF handling general cases.

The SHAP value distributions of DT and KNN are relatively narrow. Notably, the SHAP values of KNN are predominantly in the negative range, which may indicate a systematic bias in its predictions compared to the final model output. This may be due to its simple structure, which is unable to capture complex patterns, resulting in a limited contribution to the stacking model.

**Figure 7:**
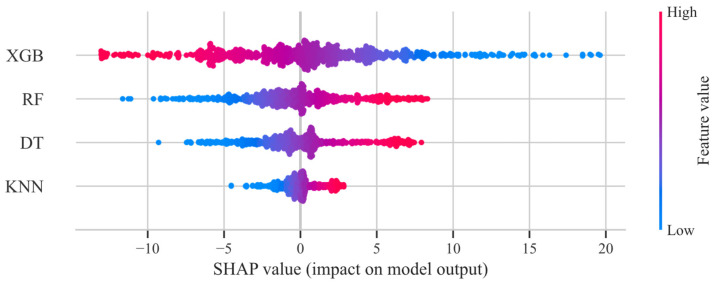
SHAP contribution analysis for the meta-learner in the second-level δ.

Similarly, for δ, as shown in Figure 7, the SHAP values of XGB exhibit the widest distribution range, spanning from −15 to 20, indicating its outstanding status. In addition, the values of the remaining three models are approximately symmetrically distributed around 0. The rest of the distribution is similar to that of G* and will not be discussed further here.

In conclusion, the contribution of the integrated model to stacking is generally better than that of the basic model. XGB is the most important base learner in the stacking model, with its predictions playing a decisive role in the outcome. Conversely, KNN may hurt the model in certain situations.

## 5. Conclusions and Prospects

This study systematically evaluated the performance of various machine learning (ML) models in predicting the rheological properties of asphalt modified with mineral powder. The primary objectives were to compare single ML models (KNN, Bayesian ridge, decision tree) with ensemble learning techniques (bagging, boosting, and stacking) and to identify the most effective approach for predicting complex shear modulus (G*) and phase angle (δ). The key findings of this research are as follows:

- Ensemble learning models, particularly the stacking model, outperformed single ML models in terms of prediction accuracy and generalization ability. The stacking model shows its superiority in *R*^2^ (0.9727 for G* and 0.9990 for δ) *MAE*, *MAPE*, and *RMSE*, demonstrating its robustness and reliability. This finding corroborates prior research [16,17,18,19,20], emphasizing the effectiveness of ensemble methods in modeling complex non-linear relationships within material-science datasets.
- SHAP analysis revealed that temperature and mineral powder type are the most significant factors affecting the rheological properties of modified asphalt. Among the mineral powders tested, the ranking of G* and δ enhancement is as follows: granite (GN) > diabase (DB) > limestone (LS). This indicates GN’s potential for improving the high-temperature stability of asphalt mixtures. This finding is consistent with the results of Xu et al. [27], who reported that mineral powder can significantly enhance the high-temperature performance of emulsified asphalt. Their study also revealed that the order of effectiveness for this improvement is as follows: GN > DB > LS.
- The SHAP analysis also provided valuable insights into the contributions of different base learners in the stacking model. XGBoost was identified as the most influential base learner, while KNN exhibited limitations in certain scenarios, highlighting the importance of model selection and integration in ensemble learning.
- The findings of this study have significant implications for the design and optimization of asphalt mixtures. The stacking model offers a data-driven approach to predict the rheological behavior of mineral powder-modified asphalt, which can aid in selecting optimal materials and improving pavement performance.

This study provides a comprehensive evaluation of ML models for predicting the rheological properties of mineral powder-modified asphalt. Future research should explore several avenues for improvement. Firstly, it is essential to expand the dataset to include a wider range of mineral powders, different types of asphalt binders, and various testing conditions. This will enhance the model’s ability to generalize across diverse scenarios and improve its predictive accuracy. Secondly, while ensemble learning models have shown promising results, integrating advanced ML techniques such as deep learning and hybrid models could further enhance prediction accuracy. Exploring the potential of these techniques in the context of asphalt rheology is a worthwhile direction for future research.

## Figures and Tables

**Table 1:** The physical properties of emulsifiers.

Property	MQ65	EM520
Physical form, 25 °C	Brown liquid	
Emulsifier type	Cation, slow-breaking, and quick-curing	
Solid content (%)	100	
Density (g/cm^3^)	1.026	1.050
Viscosity (mPa·s), 25 °C	7400	≤90,000

**Table 2:** The test results of the particle size of different mineral powders.

Property Indices	Specific Surface Area (SSA)	D10 (μm)	D25 (μm)	D50 (μm)	D75 (μm)
LS	2.108	1.249	2.282	4.557	8.423
DB	2.419	1.106	1.867	3.514	5.965
GN	1.791	1.396	2.475	5.178	8.781

**Table 3:** Performance of ML models for prediction of complex shear modulus (G*) and phase angle (δ) on datasets.

Evaluation Indicators		BR	KNN	DT	RF	XGB	Stacking
G*	*R* ^2^	0.7315	0.9973	0.9990	0.9995	0.9996	0.9727
	*MAE*	24.133	0.6751	0.6738	0.4551	0.6493	8.5964
	*MAPE*	1.5368	0.0248	0.0159	0.0113	0.0154	0.3719
	*RMSE*	32.351	3.1549	1.8610	1.3694	1.1635	10.146
δ	*R* ^2^	0.6043	0.9864	0.9982	0.9984	0.9988	0.9990
	*MAE*	1.9182	0.0885	0.0443	0.0374	0.0649	0.0409
	*MAPE*	0.0286	0.0013	0.0007	0.0006	0.0010	0.0006
	*RMSE*	2.5391	0.4712	0.1721	0.1612	0.1368	0.1276

## Data Availability

The original contributions presented in the study are included in the article. Further inquiries can be directed to the corresponding author.
